# Characterizing Microglial Morphology: Methodological Advances in Confocal Imaging and Analysis

**DOI:** 10.3390/cells14171354

**Published:** 2025-08-30

**Authors:** Juan P. Taborda-Bejarano, David B. Nowak, Fernando Chaure, Malika L. Allen, Kathryn A. Blek, Stephen Walterhouse, John R. Mantsch, Constanza Garcia-Keller

**Affiliations:** 1 Department of Pharmacology and Toxicology, Medical College of Wisconsin, Milwaukee, WI 53226, USA; jtaborda@mcw.edu (J.P.T.-B.); dnowak@mcw.edu (D.B.N.); fchaure@mcw.edu (F.C.); mallen@mcw.edu (M.L.A.); kblek@mcw.edu (K.A.B.); 2 Medical Scientist Training Program, Medical College of Wisconsin, Milwaukee, WI 53226, USA; 3 Department of Neuroscience, Medical University of South Carolina, Charleston, SC 29425, USA; walterho@musc.edu

**Keywords:** microglia, morphology, neuroimmune, analysis

## Abstract

Microglia are central to neuroimmune responses and undergo dynamic structural and functional changes in models of stress and addiction, and in response to pharmacological treatments. While transcriptomic and proteomic assays provide insights into molecular profiles, morphological analysis remains a valuable proxy for assessing region-specific microglial response. However, morphological features alone often fail to capture the full complexity of microglial function, underscoring the need for standardized methods and complementary approaches. Here, we describe a standardized imaging pipeline for analyzing microglia in the nucleus accumbens core (NAcore), integrating unbiased confocal image acquisition with precise anatomical reference points. We compare two widely used image analysis platforms—IMARIS and CellSelect-3DMorph—highlighting their workflows, output metrics, and utility in quantifying microglial morphology following treatment with adenosine triphosphate (ATP). Both tools detect well described features of microglial dynamics, though they differ in automation level, analysis speed, and output types. Our findings demonstrate that both platforms provide reliable morphological data, with CellSelect-3DMorph offering a rapid, open-access alternative for high-throughput analysis. Additionally, using software-derived parameters in principal component analysis clustering has proven useful for identifying distinct subpopulations of microglia separated by their morphology. This work provides a practical framework for morphological analysis and promotes reproducibility in microglial studies under environmental and pharmacological interventions.

## 1. Introduction

Microglia are the primary immune cells of the central nervous system (CNS) and play critical roles in maintaining neural homeostasis, responding to injury, and shaping synaptic networks. In neuropsychiatric contexts, such as stress and substance use disorders, microglia exhibit dynamic changes in both form and function [1]. Accurately characterizing these changes is essential for understanding their contributions to neuropathology. Recent literature emphasizes the need for multimodal approaches that integrate diverse data types to define microglial states more precisely and avoid overreliance on single-assay interpretations [2].

Among the techniques available for assessing microglial function, three major categories are widely utilized: transcription-based assays (transcriptomics), protein-based assays (proteomics), and morphological analyses [3,4,5,6,7]. Morphological analysis provides a valuable complementary approach, especially in in vivo and ex vivo models. Traditionally, microglia exhibiting highly ramified processes have been interpreted as homeostatic, while amoeboid (reduced ramification) forms suggest a reactive, pro-inflammatory phenotype [8,9]. However, emerging evidence reveals that microglial morphology spans a spectrum of forms that do not always align with functional activation states. As such, morphological analysis should be seen as an important—but incomplete—proxy for function [2].

To maximize the interpretability and reproducibility of morphological studies, standardization of image acquisition and analysis protocols is essential. In this manuscript, we describe a standardized confocal imaging pipeline for analyzing microglia in the nucleus accumbens (NAc), specifically in the nucleus accumbens core (NAcore), a region critically involved in reward, stress responses, and addiction pathology. Here we detail an unbiased imaging method based on anatomical reference points and high-resolution 3D confocal microscopy, followed by comparison of two widely used morphological analysis platforms: IMARIS (version Oxford Instruments, Abingdon, UK), a commercial 3D visualization and reconstruction tool, and CellSelect-3DMorph, an open-access, MATLAB-based semiautomated (version Matlab R2018a) analysis suite developed in our laboratory [10].

By highlighting methodological strengths, limitations, and analytic outputs of both platforms, we aim to provide researchers with a clear framework for selecting appropriate tools and interpreting morphological data within broader multimodal investigations. Moreover, we aim to achieve a comprehensive understanding of microglial morphological analysis, by furthering our understanding of morphology with principal component analysis (PCA) and two-step clustering, using the parameters provided by the CellSelect-3DMorph. Clustering analysis is a tool that aids in identifying different populations within the microglia present in a brain region; hence providing a better understanding of how experimental manipulations affect the neuroimmune system. However, while morphology alone cannot define microglial function, robust morphological assessment remains a critical component in the integrative analysis of microglial dynamics under stress and addiction-like conditions.

## 2. Materials and Methods

### 2.1. Animals

All housing and experimental procedures were conducted in accordance with the Medical College of Wisconsin Institutional Animal Care and Use Committee (IACUC) regulations. Animals were double or triple housed in the Biomedical Resource Center in a 12-h inverted light cycle prior to experimental manipulation. Eight female Long-Evans rats used in the experiment had ad libitum access to standard chow and water for the duration of the study. These rats were divided into two groups and given intraperitoneal injections of either adenosine 5’-triphosphate (ATP; *n* = 4; disodium salt, Millipore Sigma, Burlington, MA, USA, A7699-1G) or saline vehicle (VEH; *n* = 4). Following the injections, rats returned to their home cages for two hours. ATP was administered at a dose of 50 mg/kg, as described in [11]. Two hours after the injections, the animals were perfused, and tissue was collected.

### 2.2. Immunostaining

For the immunohistochemical staining, rats were intracardially perfused with 4% paraformaldehyde, followed by an overnight post-fixation period. Brains were sliced the following day at 100 um thickness. The tissue was stained with a macrophage and microglia marker: ionized calcium binding adaptor molecule 1 (rabbit anti-IBA-1 1:300; Invitrogen PA5-21274, Carlsbad, CA, USA) primary antibody for morphological analysis. Free floating brain slices were washed 3 times for 5 min in 1× phosphate-buffer saline (PBS); followed by a permeabilization in 1× PBS + 0.5% triton (PBST; Thermo Scientific A16046.AP, Waltham, MA, USA) for 10 min. The brains were then incubated for 1 h in a blocking solution consisting of 5% Normal goat serum (Invitrogen, PI31873) + 0.5% PBST at room temperature. After blocking step, brain slices were incubated in the blocking solution + rabbit anti-IBA-1 antibody 24 h at 4 °C on a shaker. After the primary antibody step, brains slices were washed 3 times for 10 min with 0.1% PBST, followed by a 2-h incubation in secondary antibody Alexa 568, goat anti-rabbit (1:200, Thermofisher, A78955, Waltham, MA, USA) at room temperature covered from light. The tissue was then washed 3 times in 1× PBS before mounting and cover slipping the tissue. Brain slices were mounted onto charged microscope slides and air dried before cover slipping with mounting media (Invitrogen, ProLong Gold antifade mountant, P36930).

### 2.3. Data Analysis

All data analyses were performed using GraphPad Prism 10 (Boston, MA, USA). Prior to statistical testing, datasets were assessed for normality using the D’Agostino & Pearson test. As we find that microglial morphological data were not normally distributed, we applied nonparametric tests, including the Mann–Whitney U test for two-group comparisons and the Kolmogorov–Smirnov test for distribution comparisons. Sholl analysis data were analyzed using a mixed model analysis. Note that data were analyzed at the individual cell level, which increases the apparent sample size and may overstate statistical significance. Due to the non-normal distribution, appropriate nested analyses could not be performed.

Dimensionality reduction and cluster analysis was conducted using SPSS (version 29.0.2.0) and RStudio (version R 4.5.0), with further statistical evaluation in GraphPad Prism. Clustering normalized some of the microglial morphology data and, in those instances, data were analyzed using ordinary one-way ANOVA, with Bonferroni post hoc tests applied for multiple comparisons. For non-normalized data the non-parametric Kruskal–Wallis test was followed by Dunn’s multiple comparisons. Also, Chi-square analysis was used to compare cluster frequency between groups.

## 3. Results

The following sections contain a discussion of techniques for image acquisition, morphometric processing, and data analyses, that can be applied to study microglia morphology. Through rigorous investigation of microglial morphology in models of health and disease, we aim to appreciate the consequential, and often nuanced, role of these cells in the development and persistence of neuropsychiatric disease.

### 3.1. Unbiased Image Acquisition Methodology

*A.*  Visualization of microglia

Morphological analyses rely heavily on image quality, resolution, and unbiased acquisition from a region of interest. The NAc is a subcortical brain structure known primarily for its roles in motivated behavior and as a motor-limbic interface that mediates goal-directed behaviors [12,13,14]. A subregion within the NAc, the NAcore, undergoes long-lasting synaptic plasticity in response to chronic use of addictive drugs [12,15] (e.g., heroin, cocaine, alcohol, nicotine) and stress exposure [16,17,18,19,20,21].

Microglia visualization is required to perform morphological studies, commonly using IBA-1, a cytoplasmic calcium binding protein expressed in microglia and macrophages, as a marker [22]. For the purposes of this manuscript, we refer to all IBA-1 + cells as microglia. Furthermore, there are other markers for microglia, including CD11b, CX3CR1, and CD68. Alternatively, TMEM119 and P2Y12R are markers that are exclusive for microglia. Additionally, multiple transgenic reporter lines are constructed by genetically inserting a fluorescent marker gene sequence to a gene of interest (e.g., CX3CR1^GFP^, TMEM119^GFP^, Sall1^GFP^), this insertion causes the addition of the fluorescent marker into the target protein [23,24,25]. However, these are typically mouse lines. Currently, there are two transgenic rat constructs available for microglia visualization. Two transgenic rat lines are available for microglia-specific studies: a Cre-dependent CX3CR1-ERT2 line (available from the Rat Resource & Research Center, Columbia, MO, USA), which enables inducible gene manipulation in microglia, and an IBA1-EGFP line, which allows for direct fluorescent visualization of microglia morphology [26]. IBA-1 is a good morphological marker, as the protein is distributed throughout microglial cytoplasm, thus enabling clear visualization. As a result, it has been extensively utilized as a microglial marker in various mammalian models, including rodents, non-human primates, and rabbits [27].

*B.*  Optimizing microglia visualization: slices thickness

**Figure 1:**
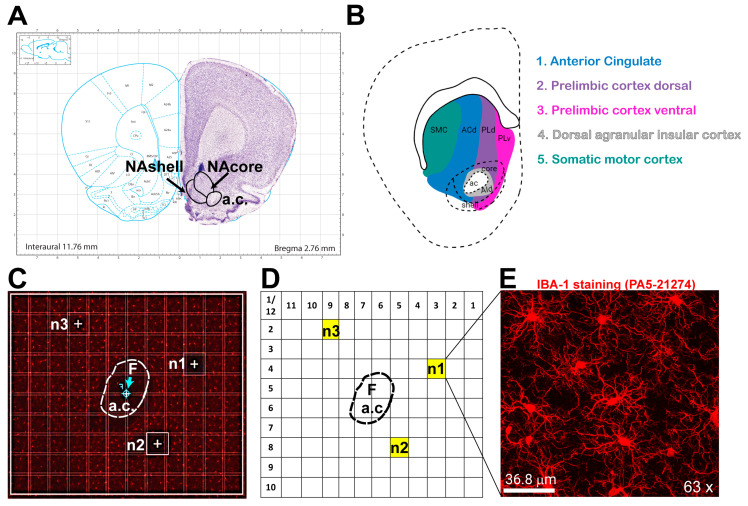
Unbiased Confocal Image Acquisition Methodology in the NAcore. An unbiased imaging method was used, capturing tissue at the same three locations across all groups, using the anterior commissure as a reference point. These positions correspond to three distinct areas within the nucleus accumbens core, each associated with different glutamatergic projections. (**A**) Paxinos’s Rat Atlas [32] showing the nucleus NAcore and nucleus accumbens shell (NAshell). (**B**) Mapping of glutamatergic projections onto different zones of the NAc, adapted from ref. [33]. (**C**) Tile scan of the nucleus accumbens at 20× magnification, with the annotated anterior commissure (a.c.), the three positions for image acquisition (n1, n2, n3), and the focal point marked as “F”. (**D**) Numbered gridlines with the drawn anterior commissure (a.c.) illustrating the specific positions of the images within the grid. (**E**) 63× magnification image of microglia in the NAcore at the n1 position.

For accurate morphological analysis, it is essential to visualize microglial cells with most—or ideally all—of their processes within the field of view. As such, 3D analyses are generally favored over 2D approaches. To obtain full 3D representations of microglia, thicker brain sections are recommended, as they allow for more complete imaging of cellular structures. We suggest sectioning the brain into 100 μm slices to facilitate this. However, previous studies have shown that 50-μm sections can also provide sufficient 3D morphological detail [28] and traditional morphological analyses have often used thinner sections, typically 30–40 μm [29,30,31]. While both image analysis platforms used in this study are capable of processing thinner sections, reduced section thickness limits the depth of the image stack. This decreases the likelihood of capturing entire microglial cells within a single field of view and may result in incomplete reconstructions. These limitations compromise the interpretability of morphological data, reinforcing the preference for thicker sections. Following microglial labeling, image acquisition should be performed using confocal microscopy, which is ideal for morphological studies due to its ability to capture high-resolution 3D images. Figure 1 illustrates our unbiased image acquisition protocol in the NAcore using confocal microscopy.

*C.*  Reference coordinates for imaging the NAcore

We developed an unbiased imaging approach that utilizes a structural reference point to reliably target regions of interest. Employing such reference points ensures consistent imaging locations across experimental groups. For imaging the nucleus accumbens core (NAcore), we use the anterior commissure (a.c.) as a landmark (Figure 1A). Guided by the Paxinos Rat Brain Atlas [32] and the work of Voorn et al. [33], we focus specifically on the NAcore and trace glutamatergic projections originating from the ventral prelimbic cortex, dorsal prelimbic cortex, and dorsal anterior cingulate (PLv, PLd, and ACd, respectively). For each subject, six total images are collected: three from predefined locations in the rostral NAcore and three from predefined locations in the caudal NAcore, as described in the following section (D). The NAcore spans an anterior–posterior (AP) range of approximately +3.00 to +0.50 mm. Within this range, we define rostral NAcore as AP +3.00 to +1.80 mm and the caudal NAcore as AP +1.80 to +0.50 mm. The tissue sections and coordinates shown in Figure 1 represent caudal NAcore example.

*D.*  Image acquisition workflow

Once cells are stained and target fields are identified, confocal imaging can begin. The following protocol outlines image acquisition using a Leica SP8 Upright Confocal Microscope (Mannheim, Germany) with the LAS X software (Version: 3.5.7.23225) suite; however, it can be adapted to other microscope models and software platforms as needed. Start by surveying the brain sections using a 5× objective (or the lowest available magnification) to locate the region of interest (ROI). Once identified, center the ROI within the eyepiece’s field of view. Switch to the 10× objective and, within the virtual image provided by the software, adjust the gain and laser intensity settings to optimize image quality and resolution across all treatment groups but blinded to conditions. Using the tile stitching function, acquire a spiral scan around the region of interest—in this case, the anterior commissure. Then, use the microscope’s focal point tool to place a marker (F) at the center of the reference point within the acquired image (Figure 1C; arrow indicates F).

For example, when imaging the nucleus accumbens (NAc), we place the focal point at the center of the anterior commissure (a.c.). Then switch to a 63× oil immersion objective, because its superior resolution and cellular detail required for morphological analysis. However, some studies have successfully used a 40× objective instead [34]. Once the 63× objective is in place, use the navigator interface to define a tile scan grid, typically a 12 × 10 layout, with each tile measuring 0.172 μm × 0.172 μm. The center of the grid (indicated by a cross within a circle) is aligned with the focal point (F) (Figure 1C). This grid ensures that image coordinates are consistent across experimental groups (Figure 1D). In the NAcore, the selected image coordinates are (3, 4), (5, 8), and (9, 2), corresponding to sampling points n1, n2, and n3, respectively. These positions were chosen because they each receive distinct glutamatergic inputs, from the PLv, PLd, and ACd regions as explained before. Maintaining consistent tile grid positions across treatment groups ensures unbiased image acquisition. Before imaging, acquisition parameters should be optimized for cell resolution and image quality. General settings that produce high-resolution images include a 1024 × 1024-pixel resolution, a scan speed of 600 Hz, line averaging of 3, and a pinhole size of 1 μm (999.89 mAU). Parameters such as gain and laser intensity often require adjustment between experiments or cohorts to minimize background noise and enhance visualization of cell processes. Importantly, all imaging parameters should be kept consistent across treatment conditions, and the investigator performing image acquisition should remain blinded to group assignments.

For Z-stack acquisition, we used a step size of 0.5 μm along the *z*-axis. Smaller step sizes improve resolution of fine cellular processes and overall cell morphology. For example, the Z-stack shown in Figure 1E is approximately 37 μm thick, capturing multiple microglia within a single field of view. This imaging approach can be readily adapted to other brain regions of interest. Notably, regions such as the medial prefrontal cortex and ventral tegmental area—both implicated in stress and addiction—are also relevant targets for microglial analysis [35,36].

### 3.2. Morphological Analysis Tool Comparison

Morphological analyses of microglia typically focus on the ramification states of the cells; historically these states have been defined by calculating the quantities and lengths of branches, with more and longer branches indicating more ramified microglia compared to amoeboid microglia, which have fewer and smaller branches [2,37,38]. In the past decades, multiple platforms have been developed for morphological analyses; each with pros and cons, making the selection of the proper tool challenging. Prior to selecting an analysis tool, a criterion must be set to include or exclude cells for the analysis. A good criterion for 3D analysis of microglia is to exclude incomplete cells (i.e., cells that have less than 80% of its soma and projections within the *xyz* planes of the image), as morphology cannot be accurately assessed in those cells. Following criterion selection, an analysis tool must be selected. A commonly used software is IMARIS (Oxford Instruments, Abingdon, UK), as it is capable of 3D analysis.

*A.*  Microglial morphological analysis using IMARIS

**Figure 2:**
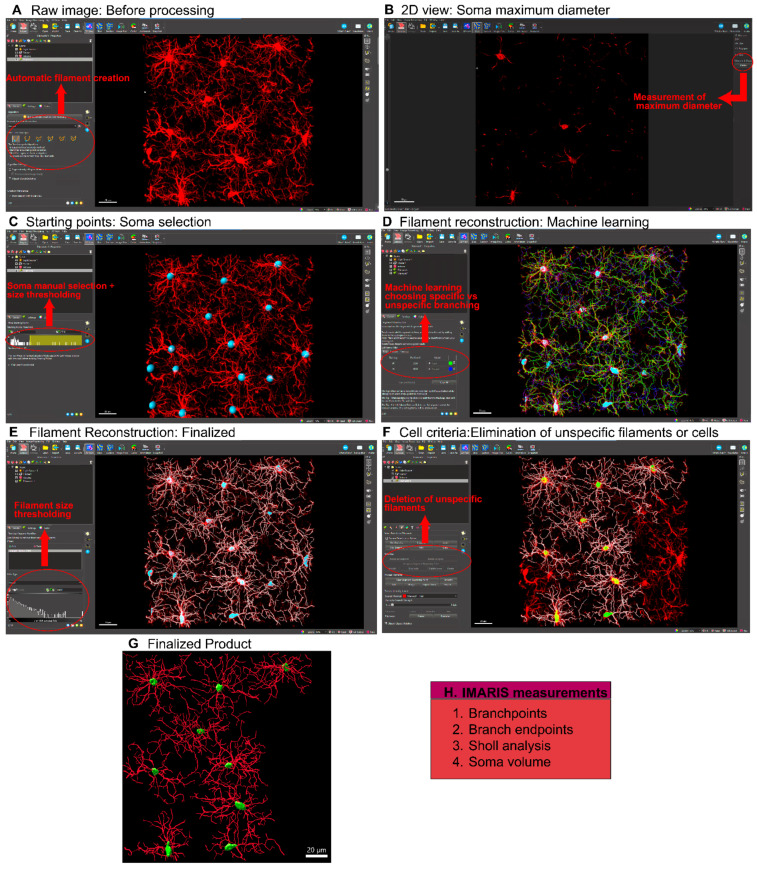
Microglial Analysis Workflow Using IMARIS Software. The images were analyzed with IMARIS 10.10, utilizing the filament creation function to extract detailed morphological data. (**A**) Begin by selecting the filament creation function and applying the automatic creation option. (**B**) Next, choose the largest diameter of the cells. (**C**) Once the diameter is selected, the software will automatically populate the soma size within the image. (**D**) IMARIS employs machine learning to distinguish between specific and non-specific branching during reconstruction. This step is crucial as the parameters from this machine learning process can be applied to all other images. (Green indicates a detected branch surface). (**E**) Use volume thresholding to remove small, non-specific projections before completing the reconstruction. (White indicates selected branch surface). (**F**) After reconstruction, remove any incomplete cells whose morphology cannot be accurately measured. (White indicates selected branch surface and yellow indicate cell bodies selected). (**G**) The final output consists of cells whose morphology can be accurately assessed. (**H**) IMARIS provides a broad range of statistical measurements, with the most relevant for morphological analysis being branch points, branch endpoints, sholl analysis, and soma volume.

IMARIS is an application that aids in the visualization of confocal images in 3D and has options that enable reconstruction and measurement of cell morphology and colocalization, as well as protein and cell quantification (in addition to a range of other applications). Here, we will focus on the morphological analysis features of IMARIS. Cell morphology can be analyzed using the IMARIS cell surface and filament functions which reconstruct the cells and define characteristics that relate to morphology. Cell surface creation permits measurement of cell and soma volumes, while filament creation enables measurement of branch endpoints, branchpoints, and branch length (sum), and Sholl analysis-based analysis of branch complexity, among other measures. We recommend using the filament creation function, as it measures more morphological characteristics that allow for accurate analyses compared to the cell surface creation function. Figure 2 presents a stepwise summarized protocol for filament creation in IMARIS. The filament creation function in IMARIS has an automatic system (Figure 2A) by which the soma diameter and fluorescent thresholding are set (Figure 2B). However, the microglial somas must be manually positioned (Figure 2C). Once this is done, IMARIS machine learning will reconstruct the branches of the microglia (Figure 2D). With machine learning, it is possible to teach IMARIS to accurately define branches. IMARIS will store this information in parameter files so that it can be applied to future images. Once the initial reconstruction is complete (Figure 2E), exclude cells that are not within the criteria (Figure 2F) and eliminate any filaments that are not connected or do not originate from the same cell to obtain the finalized image (Figure 2G). Note that IMARIS includes a batch processing feature, which is a useful tool for quickly processing multiple images. However, for this specific analysis, each image must be individually reviewed to accept or remove incomplete cells based on defined criteria. As a result, we chose to manually process all images from the outset, rather than use batch processing followed by manual editing. Although IMARIS provides excellent visualization of the cells as well as accurate reconstruction, the time required for this process is high relative to some other platforms.

*B.*  Microglial morphological analysis using CellSelect-3DMorph

In 2018, an open-access, semi-automated microglial analysis tool called 3DMorph was introduced [34]. The MATLAB-based code had multiple advantages that encouraged us to update and optimize the base code [10]. Due to the extensive modifications made, we rebranded the improved version as CellSelect-3DMorph 1.0 (DOI: 10.5281/zenodo.14159877). A key improvement over the original version is its compatibility with current MATLAB releases, along with the availability of a standalone executable that does not require a MATLAB license. Additionally, the updated version offers enhanced efficiency through faster processing and provides users with the ability to selectively include or exclude specific cells during analysis. Both the original and updated versions are freely available on the Garcia-Keller laboratory’s GitHub public repository: https://github.com/CGK-Laboratory/CellSelect-3DMorph (version 2.0, 13 November 2024).

**Figure 3:**
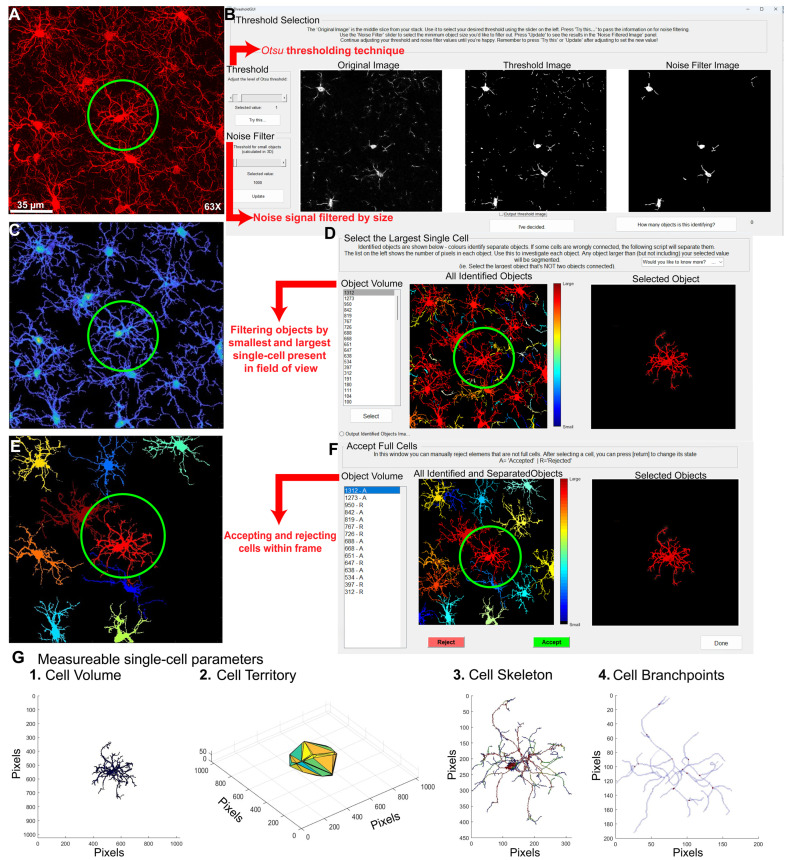
Microglial Analysis Workflow Using CellSelect-3D Morph. (**A**) Raw confocal image of microglia stained with IBA-1. (**B**) The first step is to apply the Otsu thresholding technique, adjust the fluorescent intensity for reconstruction, and select a noise filter to reduce background signaling. (**C**) Reconstructed image using the chosen Otsu threshold. (**D**) At this stage, the entire image is reconstructed, and both the largest and smallest single cells are selected to filter out non-cellular structures and separate reconstructions where two cells are considered as one. (**E**) 2D image showing the selected cells. (**F**) This window allows for the selection of cells to be measured, filtering for incomplete cells. (**G**) Reconstructed single-cell images (i.e., circled cell) outputted by the software. Panels (**1**–**4**) show different reconstructions representing the various parameters and measurements provided by the software. Panel (**C**,**D**,**F**) have cells represented in different colors defined by the pixel size of each reconstructed cell.

CellSelect-3DMorph is a semi-automatic tool in which cell reconstruction is performed automatically, but the user manually guides key decisions. After confocal imaging (raw image, Figure 3A), an Otsu threshold is applied to each image to distinguish foreground (cell signal) from background (Figure 3B) [39]. In the same step, a noise filter removes small artifacts or non-cellular objects, with the minimum object size defined by the user. The software then prompts the user to identify the largest individual cell in the image (Figure 3D). This step acts as a quality control checkpoint, as thresholding may occasionally merge multiple cells into one object. By identifying a correctly sized single cell, the software can exclude any larger merged artifacts. Similarly, users identify the smallest object that qualifies as a complete cell, enabling the software to exclude smaller, non-cellular debris. Cells within this defined size range are then selected for full analysis (Figure 3F). After cell selection, CellSelect-3DMorph generates a complete reconstruction of each selected cell (Figure 3E), followed by the creation of 3D skeletons and reconstructions (Figure 3G). The software also produces a Microsoft Excel™ spreadsheet containing all quantitative morphological measurements. It is important to note that some entries in the final data table—such as branchpoints, endpoints, or branch length values—may appear as zero. These occasional errors are likely due to rendering limitations that may arise from either hardware or software constraints. Additionally, images with high background fluorescence may not render well, as CellSelect-3DMorph is sensitive to background signal. While image deconvolution can improve results, it must be uniformly applied to all images for consistency.

CellSelect-3DMorph will quantify several key morphological parameters: cell volume, cell territory, ramification index, branchpoints, branch endpoints, average branch length and minimum and maximum branch length. Cell volume refers to the volume of the fluorescent pixels encompassing the cell (Figure 3(G1)). Cell territory is the maximum expansion of the cell measured by a polygon surrounding the cell (Figure 3(G2)). The ramification index is calculated by territorial volume divided by cell volume. Higher values suggest a more ramified cell, where the maximum projection area significantly exceeds the cell’s volume, while lower values indicate an amoeboid shape, with the projection area closely matching the cell volume. Branchpoints quantify the bifurcating points in process branching (Figure 3(G3,G4)), while endpoints quantify all branches. Branchpoints, branch endpoints, minimum and maximum branch length, and average branch length are measurements of cell complexity which have been traditionally used to assess morphology in an indirect manner. However, they are useful for assessing the complexity of cell morphology (similar to IMARIS). Both CellSelect-3DMorph and IMARIS are similar in their morphological measurements. However, CellSelect-3DMorph is faster than IMARIS for image analysis and can provide a ramification index measurement, while IMARIS can also provide the volume of the soma. Nonetheless, both analyses are useful for determining microglia morphology.

### 3.3. Comparison Between IMARIS and CellSelect-3DMorph Outputs

**Figure 4:**
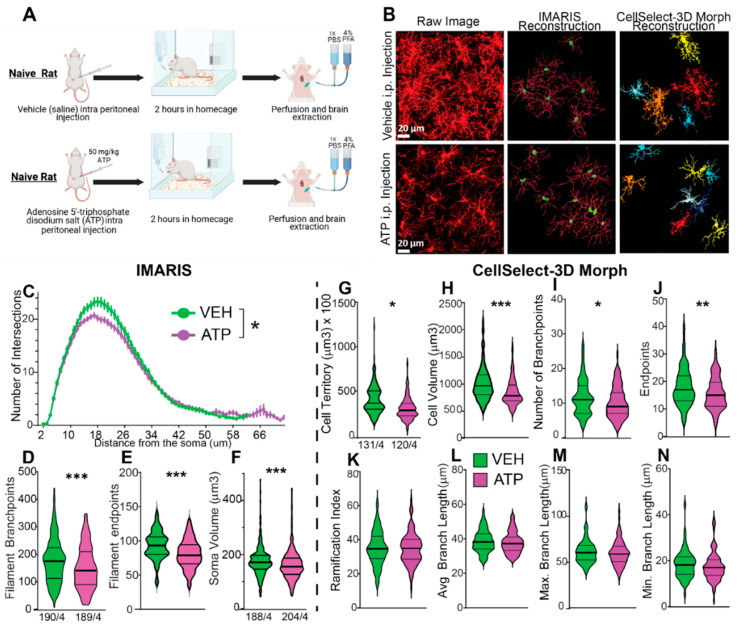
Comparative Analysis of the Same Dataset Using CellSelect-3D Morph and IMARIS Software. The dataset was collected from naïve female rats treated with either vehicle (saline) or ATP disodium salt (50 mg/kg) 2 h before perfusion and tissue collection. Comparable data were obtained from both software programs used. (**A**) Timeline detailing the treatment with vehicle and ATP i.p. injections in naïve rats, followed by the perfusion protocol. (**B**) *Left panel*: Raw images from confocal microscope of microglia stained with IBA-1 in vehicle and ATP-treated rats. *Middle panel*: Reconstruction of microglia using IMARIS software. *Right panel*: Reconstruction of microglia using CellSelect-3D Morph software. Figures (**C**–**F**) display parameters obtained using IMARIS software. Specifically, after ATP treatment panel (**C**) shows a reduced number of intersections (95% Confidence interval: [0.1970, 1.734]), (**D**) depicts reduced the total number of filament branch points (r effect size = −0.1605, 95% Confidence interval median difference (CImd): [−41.00, −12.00]), and (**E**) illustrates reduced the total number of filament endpoints (r = −0.1622, 95% CImd: [−21.00, −6.000]). Panel (**F**) shows a reduction in cell body volume after ATP treatment (r = −0.4791, 95% CImd: [−35.00, −18.00]). Figures (**G**–**N**) display parameters obtained using CellSelect-3D Morph software. Specifically, after ATP treatment panel (**G**) shows reduced cell territory (r = −0.1469, 95% CImd: [−7343, −680.8]), (**H**) reduced cell volume (r = −0.2110, 95% CImd: [−166.1, −44.82]), (**I**) reduced number of branchpoints (r = −0.1982, 95% CImd: [−3.000, 0.000]), and (**J**) number of endpoints (r = −0.1657, 95% CImd: [−4.000, −1.000]). Panel (**K**–**N**) show no changes in ramification index, average branch length, and min and max branch length. Data are shown as median and quartiles. Figure (**C**) was analyzed with Mixed-effect analysis indicating a * *p* < 0.0001 difference between ATP and Veh. Figures (**D**–**N**) were analyzed with non-parametric test Mann–Whitney indicating * *p* < 0.05 difference between ATP and Veh, ** *p* < 0.01 difference between ATP and Veh, and *** *p* < 0.001 difference between ATP and Veh. Figures (**D**,**F**,**G**) show the number of microglia/number of rats. Additional analysis was performed with non-parametric test Kolmogorov–Smirnov on panels (**D**–**N**) indicating * and ** *p* < 0.01 difference between ATP and Veh, and *** *p* < 0.001 difference between ATP and Veh (Kolmogorov–Smirnov test showed no difference on panel (**J**). Panel (**A**) was performed using BioRender.com. The right images in panel (**B**) show CellSelect-3Dmorph reconstructions, where each cell is represented in a different color based on its pixel size.

To demonstrate the utility of the software for microglial morphological analysis, we present data analyzed using both tools, highlighting their differences and similarities. The experiment involved artificially stimulating a response from microglia through an intraperitoneal injection of adenosine 5’-triphosphate (ATP) disodium salt. ATP disodium salt induces a microglia response by activating purinergic receptors, including P2X and P2Y receptor subtypes [40]. This experiment utilized 10-week-old naïve female rats, divided into two groups: one group received intraperitoneal (i.p.) injections of ATP diluted in ultrapure water, while the control group received vehicle (saline) injections. ATP was administered at a dose of 50 mg/kg, a concentration previously shown to activate microglia in rats [11]. Following the injections, rats were returned to their home cages for a period of two hours, after which rodents were perfused, and tissue was collected. Rats were perfused with 4% paraformaldehyde (PFA) followed by a 24-h post fixation also in 4% PFA (Figure 4A). The NAcore was imaged as shown in Figure 1 and analyzed according to the workflows illustrated in Figure 2 and Figure 3. Importantly, identical inclusion and exclusion criteria were applied across both software platforms during analysis. Figure 4B displays representative images from each condition, including raw images as well as those reconstructed using IMARIS and CellSelect-3DMorph for each group. Given the dynamic nature of microglia and emergence of distinct subpopulations [2,38,41], the morphological measurements obtained from both software platforms exhibit non-normal distributions, requiring the use of non-parametric statistical methods for valid analysis. The data were analyzed using the non-parametric Mann–Whitney rank-sum test. To further validate the findings, the Kolmogorov–Smirnov test for cumulative distribution comparison was also applied. Both statistical tests yielded consistent results. Notably, data were analyzed at cell level, which inflates sample size and overestimates statistical significance. Sholl measurement data satisfied normality assumptions, enabling the use of parametric statistical analysis. Accordingly, a mixed-effects parametric test was applied to assess the Sholl data. The analysis, conducted with each software platform, indicates that ATP treatment reduced ramification, the number of branchpoints and the overall cell size of microglia, compatible with the transition to an amoeboid-like morphology. Specifically, IMARIS analysis (Figure 4C–F) revealed reductions in Sholl intersections, branchpoints, endpoints, and ramification in ATP-treated animals. Similarly, CellSelect-3DMorph analysis (Figure 4G–N) showed decreased cell volume, territory, branch number, and endpoints following ATP exposure.

While both tools yielded comparable biological results, CellSelect-3DMorph offered a higher-throughput option due to its semi-automated workflow. On average, CellSelect-3DMorph processed approximately 6 images per hour, compared to 1–2 images per hour with IMARIS. This difference is partly due to IMARIS’s largely manual workflow, where cells must be selected, reconstructed, and edited individually to meet selection criteria. In contrast, CellSelect-3DMorph automates much of this process, requiring only limited user input for filtering and cell selection. Note that throughput rates can vary depending on hardware performance and the specific criteria used by the analyst. For example, IMARIS detected 60–80 more cells than CellSelect-3DMorph (Figure 4F,G). IMARIS enables analysis of all visible cells by offering flexible segmentation controls, allowing users to manually adjust and include cells that might be missed due to image variability. In contrast, CellSelect relies on automated reconstruction, segmentation and erosion of each cell, which can occasionally misidentify or exclude cells, especially in images with high background noise or reconstruction artifacts, due to rigid thresholding and erosion parameters.

Overall, our findings demonstrate that both IMARIS and CellSelect-3DMorph are effective software tools for microglial morphology analysis, yielding consistent and biologically relevant outcomes. CellSelect-3DMorph provides a faster, semi-automated alternative, while IMARIS offers more detailed manual control and higher total cell detection.

### 3.4. Cluster Analysis Pipeline

While microglial morphology remains a valuable tool for assessing microglial states, it has inherent limitations. Morphology alone may not reliably predict function, static imaging cannot capture dynamic behavior, and resolving distinct subpopulations is challenging. Additionally, morphological data often follow non-parametric distributions, limiting the interpretability of traditional analyses. To overcome these challenges, we implemented a cluster analysis pipeline using principal component analysis (PCA) followed by a uniform manifold approximation and projection (UMAP) analysis with K-means clustering. PCA and UMAP are dimensionality reduction techniques- linear and non-linear, respectively. The PCA approach enables the identification of distinct microglial subpopulations based on the morphological parameters mentioned above. UMAP analysis with K-means clustering identifies unsupervised clusters or groupings, by revealing shifts or biases in subpopulation distribution in response to experimental manipulation. This method offers a more nuanced understanding of microglial state, surpassing the insight provided by single-parameter or mean group comparisons.

A. Cluster analysis workflow

Cluster-based analysis of microglial morphology was carried out in three primary steps: data normalization, PCA and UMAP analysis, and then K-means clustering. First, data were normalized using Z-score transformation, calculated as *z* = (*x* − *μ*)/*σ*, where *x* is the raw value, *μ* is the mean, and *σ* is the standard deviation. This standardization ensures that all variables are on the same scale, enabling effective dimensionality reduction during PCA and UMAP. For normalization, data were organized in Excel with columns representing morphological parameters and rows corresponding to individual cells, labeled by animal and treatment groups. Z-scores were calculated with both groups pooled together using the pooled mean and standard deviation. Second, PCA was performed using SPSS to reduce the dataset into a smaller set of uncorrelated variables that capture the majority of variance in the data. This transformation allows for clearer identification of major patterns and relationships among morphological parameters measured. With the parameters given by the PCA, we then performed a UMAP analysis to project highly dimensional data into a 2D space, while maintaining the overall structure of the data. Lastly, K-means clustering was applied to identify groups of similar data points within the reduced space. This allowed us to define distinct clusters which represent the different microglial morphologies within the dataset. This approach reduced the noise from high-dimensional data, enhanced interpretability, and supported the classification of microglia morphology into distinct subpopulations.

**Figure 5:**
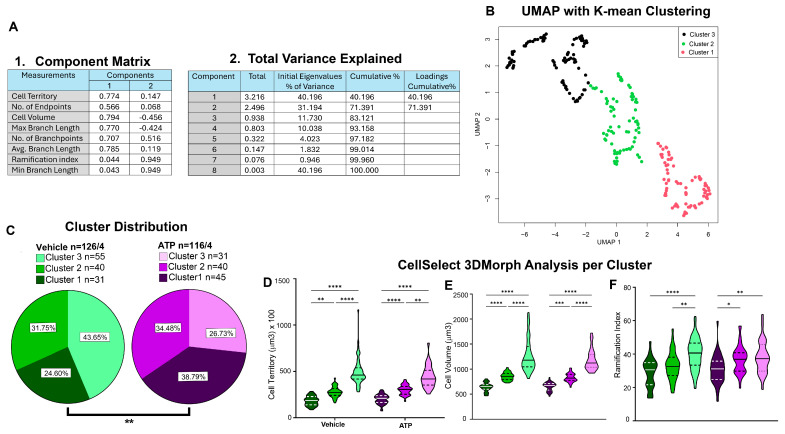
Identification of Morphological Subpopulations Using CellSelect-3DMorph Clustering. This analysis approach provides deeper insights into microglial morphological diversity. (**A**) 1. Principal component analysis (PCA) loading matrix displaying all eight morphological parameters from CellSelect-3DMorph, with corresponding loading values per principal component. 2. Variance explained by each PCA component, showing that the first two components account for 71.51% of the total variance. (**B**) Uniform manifold approximation and projection (UMAP), a dimensionality reduction graph with K-mean clusters superposed. (**C**) Cluster distribution across experimental groups, demonstrating that ATP treatment significantly alter microglial morphological distribution compared to VEH (Chi square: *p* = 0.0118; Chisqr = 8.79; df = 2) (*p*). (**D**–**F**) Comparison of specific morphological features: cell territory (r = −0.8417, *p* < 0.0001), cell volume (r = −0.8398, *p* < 0.0001), and ramification index (F_(5236)_ = 10.53, *p* < 0.0001), between clusters and experimental conditions. Data is shown in quartiles and medians. Panel (**D**–**E**) were analyzed using Kruskal–Wallis non-parametric analysis, showing a difference between clusters within the ATP and VEH groups ** *p* < 0.01, *** *p* < 0.001 and **** *p* < 0.0001. Panel (**F**) was analyzed using ordinary one way-ANOVA indicating differences/between cluster within ATP and VEH groups * *p* < 0.05, ** *p* < 0.01 and **** *p* < 0.0001. Panel (**C**) shows the number of cells analyzed in each group/animal, and the number of cells in each cluster.

We conducted cluster analysis with the dataset from Figure 4. All data were pooled and analyzed using the methodology described above. Pooling ensured that cells from both groups (ATP and VEH) were subjected to the same clustering criteria. To initiate the analysis, all morphological parameters provided by the software including cell territory, cell volume, ramification index, number of branch points, etc., were normalized using Z-score transformation. PCA revealed that all eight parameters demonstrated high quality, indicating they accounted for a substantial portion of the variance in the dataset (Figure 5A, Table 1), and the first two principal components together explained 71.39% of the total variance (Figure 5A, Table 1). UMAP analysis combined with K-means clustering revealed three distinct cluster profiles encompassing all cells (*n* = 242) (Figure 5B). Importantly, the clustering was performed in an unsupervised manner and resulted in three different clusters. Each cluster demonstrated distinct cellular profile independent of their treatment group: Cluster 1 (*n* = 76) was characterized by amoeboid morphology, Cluster 2 (*n* = 80) showed intermediate morphology with more variable shape, and Cluster 3 (*n* = 86) displayed more ramified forms.

Subsequently, data were stratified by VEH and ATP treatment groups. We first observed frequency differences between VEH and ATP treatments using Chi-square analysis. Figure 5C shows significant variation in the microglial population distribution between VEH and ATP groups, elucidating redistribution of cells along the clusters. The difference in cluster distribution between the two treatment groups suggests that the observed variations in microglial properties from Figure 4 may be due to shifts in microglial population composition. Prior to analyzing morphological features, a normality test was conducted to assess data distribution. Results indicated that some parameters conformed to a normal distribution, while others did not. Specifically, cell territory and cell volume (Figure 5D,E) deviated from normality, whereas the ramification index (Figure 5F) met the criteria for normal distribution. Accordingly, appropriate parametric or non-parametric statistical tests were applied during the analysis. Clustered microglia morphology data revealed that clusters within the VEH and ATP treatment groups exhibited similar overall behavior. A non-parametric Kruskal–Wallis test indicated significant differences among clusters 1, 2, and 3 in terms of cell territory and volume (Figure 5D,E), regardless of treatment condition. Likewise, a parametric one-way ANOVA test showed that ramification index measurements displayed no direct differences between VEH and ATP groups when comparing corresponding clusters. However, within-group comparisons revealed significant variation in ramification index among clusters in both VEH and ATP conditions (Figure 5F). Specifically, VEH clusters 1 vs. 3 and 2 vs. 3 differed significantly, as did ATP clusters 1 vs. 3 and 1 vs. 2.

Taken together, these findings reinforce the distinct microglial profiles identified through K-means clustering—cluster 1 (amoeboid), cluster 2 (intermediate), and cluster 3 (ramified). The absence of differences in the morphological parameters across treatment conditions indicates that the statistical differences observed are due to changes in the number of cells that correspond to each cluster and not the values of the parameters. This clustering approach highlights the heterogeneity of microglial responses and deepens our understanding of how ATP treatment modulates morphology at the subpopulation level. Overall, classifying microglial populations based on morphology allows for improved characterization of the forms observed under specific conditions, offering insights into which microglia population may be driving particular effects.

## 4. Conclusions

Morphological analysis, while often used as an indirect measure of microglial activation, provides only a partial view of functional state. Despite its limitations, it remains a valuable approach due to its compatibility with ex vivo imaging, regional specificity within the brain, and the accessibility of open-source software tools.

To enhance the reproducibility and reliability of morphological assessments, we developed a standardized confocal imaging protocol anchored to anatomical landmarks within the NAcore. This approach ensures consistent, unbiased image acquisition across samples. Additionally, we compared two image analysis platforms, IMARIS and CellSelect-3DMorph, highlighting their respective strengths and limitations. IMARIS enables robust 3D reconstructions and flexible analysis, though it is time- and labor-intensive. Conversely, CellSelect-3DMorph is open-source, faster, and semi-automated, offering unique features such as the ramification index, and demonstrated comparable performance in detecting ATP-induced morphological changes.

In addition, we compiled a table summarizing widely used morphological analysis tools (Table 1). A key trend identified in the literature is the field’s shift away from a traditional dichotomous classification of microglial morphology toward approaches that emphasize the identification of distinct subpopulations based on subtle morphological differences. This shift reflects both the complexity of microglial phenotypes and the time-intensive nature of detailed morphological analyses, which aligns with the themes of this work.

Still, morphological features alone are insufficient to definitively define microglial phenotypes or infer function. The integration of clustering analysis with morphological data offers a more refined approach, enabling the identification of distinct microglial subpopulations and revealing subtle, yet biologically relevant, heterogeneity in response to stimuli such as ATP. This adds an important layer of interpretability that morphology alone cannot provide.

Looking ahead, a comprehensive understanding of microglial behavior under stress, drug exposure, or environmental changes, future studies must integrate morphological and clustering analyses with molecular profiling techniques, including transcriptomics and proteomics. Nevertheless, the standardization of imaging and analysis protocols, combined with validated, accessible software tools, marks a critical step toward more reproducible, nuanced, and functionally meaningful microglial research in both neuropsychiatric and neuroinflammatory models.

## Figures and Tables

**Table 1:** Summary of widely used morphological analysis tools or methodological pipelines. Comparison of Microglial Morphology Analysis Tools and Visualization Techniques.

Tool/Method	Reference/Source	Pros	Cons	Notable Features/Notes
IMARIS	Proprietary software	- Robust 3D rendering - Versatile analysis modules - Good documentation	- Expensive - Time/labor-intensive - Requires training	Industry standard for 3D imaging; suited for detailed, high-res analysis
CellSelect-3DMorph	In-house	- Open-source - Faster than IMARIS - Includes ramification index	- MATLAB required - Limited customization	Optimized for 3D microglial analysis in confocal z-stacks
MIC-MAC	Salamanca et al., 2019 [42]	- Open-source - Combines clustering with morphology - Good for 4D data	- May need classifier training - Less flexible for custom metrics - MATLAB required	Identifies subpopulations based on morphology with clustering
3DMorph	York et al., 2018 [34]	- Easy to use - Open-source - Works on 2D/3D skeletons	- Older MATLAB version required - Assumes well-isolated cells	Widely adopted; suitable for standard morphometric analysis
Microglia morphology quantification tool (MMQT)	Heindl et al., 2018 [43]	- High-throughput - Unsupervised and automated analysis	- Time intensive - Image preprocessing needed	Suited for dynamic changes in microglia without introducing bias
MORPHIOUS	Silburt & Aubert 2022 [30]	- Measures and classifies microglia on a whole brain region - Scalable and modular	- Uses strict classification and prior machine training - Not beginner-friendly	Classifies activation states based on morphological complexity
MorphOMICs	Colombo et al., 2022 [44]	- Broad analysis and classification of microglia - Quantifies complexity and state	- Not a software but a topological data analysis approach - Very time intensive with classifier machine learning - Multiple dependencies and software needed	Ideal for large datasets and custom pipelines
MicrogliaMorphology (ImageJ tool) and MicrogliaMorphologyR (R package)	Kim et al., 2024 [29]	- Standardized IHC-based pipeline - User-friendly workflow - Vast diversity in microglial measurements	- Pipeline of analysis instead include multiple software - Manual input for some steps	Good analysis pipeline for classification and identification microglial subpopulations
FracLac and AnalyzeSkeleton (ImageJ plugin)	Young & Morrison 2018 [45]	- Fractal dimension analysis - Skeleton-based length/branching - Easy to use	- Only provides complexity (no cell-level metrics) - 2D only	Great for comparing activation via complexity (e.g., resting vs. activated) and measuring branching, length, and endpoints
Inflammation-Index	Clarke et al., 2021 [46]	- High-throughput - Motion tracking and morphometry	- Best for time-lapse - Preprocessing and prior machine learning required	Suited for dynamic, in vivo imaging studies

## Data Availability

The original contributions presented in this study are included in the article. Further inquiries can be directed to the corresponding authors. The raw data supporting the conclusions of this article is publicly available in https://doi.org/10.5281/zenodo.16921032 (13 November 2024) data repository.
